# Heroism and paramedic practice: A constructivist metasynthesis of qualitative research

**DOI:** 10.3389/fpsyg.2022.1016841

**Published:** 2022-11-07

**Authors:** Nigel Rees, Julia Williams, Chloe Hogan, Lauren Smyth, Thomas Archer

**Affiliations:** ^1^Pre Hospital Emergency Research Unit, Welsh Ambulance Services NHS Trust, Institute of Life Sciences, Swansea University, Swansea, United Kingdom; ^2^School of Health and Social Work, University of Hertfordshire, Hatfield, United Kingdom; ^3^School of Medicine, University Hospital of Wales, Heath Park, Cardiff, United Kingdom

**Keywords:** paramedic, heroism, metasynthesis, ambulance, hero

## Abstract

**Objectives:**

We aimed to identify, appraise, and synthesise the qualitative literature to develop theory on heroism and paramedic practice.

**Hypothesis/research question:**

What does published literature tell us about heroism and paramedic practice?

**Setting:**

Paramedics and other healthcare workers (HCWs) faced an outpouring of public support for them early in the COVID-19 pandemic which brought into focus the relationship between them and society, where they are portrayed as heroes.

**Participants:**

We conducted a metasynthesis using Evolved Grounded Theory and procedural guidelines of Noblit and Hare to guide analysis. Preferred Reporting Items for Systematic Reviews and Meta-analysis Protocols (PRISMA-P) guidelines were also applied.

**Results:**

151 papers were retrieved and eleven included in the final sample. Studies were moderate to very low quality, involving a wide range of methodologies and settings; none specifically explored heroism and paramedic practice. The following interrelated themes were constructed on heroism and paramedic practice: (a) *Myth, Folk law, and storytelling in heroism and paramedic practice* (b) *The epic journey of heroism and paramedic practice* (c) *Heroes and Zeroes: The fluctuating Societal Value in heroism and paramedic practice* (d) *Politicisation, and objectification in Heroism and Paramedic practice.*

**Conclusion:**

Paramedics have long been characterised as heroes, but this may not reflect their everyday experiences. Heroism in paramedic practice can provide scripts for prosocial action, inspiring others, and leading to more social heroic actions. Paramedics may however be ambivalent to such heroism narratives, due to politicisation, and objectification in the media and society. This metasynthesis is only one of many possible constructions of heroism and paramedic practice and is the first point in making sense of and developing theory on heroism and paramedic practice.

**Study registration:**

PROSPERO: CRD42021234851.

## Introduction

Exceptional demands have been placed on paramedics and other HCWs globally during the COVID-19 pandemic, bringing into focus the relationship between them, and society in an unprecedented way, and resulting in an outpouring of public support. Across the world paramedics and other HCWs have been portrayed as heroes; in the United Kingdom, public buildings have been lit up in NHS blue, the hashtag #NHSHeroes has trended on social media, and a very visible campaign of NHS Heroes was promoted within society by charities, media, government and the wider public (NHS Heroes, 2022).

Many definitions and differing views of heroism exist within the literature; however, key factors include the risk of potential physical or social harm, willingness to accept the consequences of action, and acting without the expectation of gain, and for benefit of others (Rankin and Eagly, 2008; Franco et al., 2011; Kohen, 2013). The notion of heroism and paramedic practice recently emerged in a study conducted by members of our team exploring paramedic experiences of providing care in Wales (United Kingdom) during the 2020 COVID-19 pandemic (Rees et al., 2021). The outpouring of public support and heroization of paramedics revealed complex discourses surrounding their relationship with society and tragic personal and professional choices being made. Cox (2020) also reflected such discourse, arguing that the heroism narrative within the COVID-19 pandemic may be damaging, as it stifles meaningful discussion about the limits to healthcare professionals’ duties and treatments, fails to acknowledge the importance of reciprocity, and can have negative psychological effects on workers themselves through its implication that all healthcare workers have to be heroic.

Our previous study resulted in an Evolved Grounded Theory of the tragic choices paramedics experienced in providing care during the COVID-19 pandemic and found that whilst the public held them up as heroes, paramedics did not consider themselves within this characterization, but rather felt they were just doing their job (Rees et al., 2021). Tangherlini (2000) found similar, but far more cynical and self-deprecatory accounts from paramedics of such heroic status, which countermands the public, and media representations of them as silent heroes just doing their job. Rather, Tangherlini (2000) found that Paramedics tended to present themselves as anti-heroes, quick for a sardonic quip. Heroism in paramedic practice is therefore complex. Theory is essential for the development of professional knowledge and important in defining professional identity (Marrs and Lowry, 2006), but despite this, there is a significant lack of attention to heroism in paramedic practice literature. Therefore, to make sense of this complexity, research is needed, especially qualitative studies which may reveal important insights, and understandings Therefore through this metasynthesis we aim to identify, appraise and synthesize the qualitative literature in order to develop theory on heroism and paramedic practice. These insights are important and will support more practical purposes of informing paramedic policy, practice, and education.

## Materials and methods

We adhered to our published plan of investigation as outlined in the study protocol and in accordance with the Preferred Reporting Items for Systematic reviews and Meta-Analysis Protocols (PRISMA-P) guidelines (Rees et al., 2021).

We used the following criteria to select studies for analysis:

1. The expressed *a priori* purpose of the study was to examine paramedic, nurse, or doctor
2. A focus on heroism or hero/heroine or for paramedics
3. Ambulance OR emergency OR pre-hospital care OR EMS
4. The studies were conducted using qualitative methods OR Narrative OR Opinion pieces.

Searches were undertaken of the databases CINAHL®, MEDLINE®, OVID ®, SSCI – Social Sciences Citation Index (*via* the Web of Science), Scopus ®, and Psych INFO®. Our search strategy will include the search terms of (“Hero” OR “Heroic” OR “Heroism” OR “Heroine”) AND (“Paramedic” OR “Emergency carer” OR “EMT” OR “Nurse”) OR (“ambulance” OR “Emergency Medical Service” or “EMS” OR “pre Hospital” OR “Emergency Department”). Limitations were applied to the search to return results in the English language, but no limits on publication dates included.

Each citation title and abstract was reviewed independently by two reviewers to identify articles likely to be eligible. Any disagreements between reviewers were resolved through discussion. The two reviewers independently applied the Critical Appraisal Skills Program (CASP) quality assessment tool for qualitative studies.

The epistemological basis of the study follows the constructivist paradigm of inquiry, which sees the world as constructed, interpreted, and experienced by people in their interactions with each other and with wider social systems (Denzin and Lincoln, 2000). We drew on the Evolved Grounded Theory Methodology (GTM) of Strauss and Corbin (1990) which reflects early social sciences approaches to meta-ethnography and relies on conceptual coding and construction of new theory (Timulak, 2009). We also used Noblit et al. (1988) procedural guidelines to guide the analysis.

### Ethics

The protocol was registered with the International Prospective Register of Systematic Reviews PROSPERO 2021, registration number CRD42021234851. We also prospectively disseminated our protocol publicly through publication in a peer-reviewed journal and *via* conference presentations. This metasynthesis is a retrospective study drawing on publicly available data; it does not involve NHS staff or patients and therefore NHS Research Ethical review was not required within Health Research Authority guidance (HRA, 2020).

### Patient and public involvement

No public or patient involvement due to the nature of the study.

### Results

**Figure 1:**
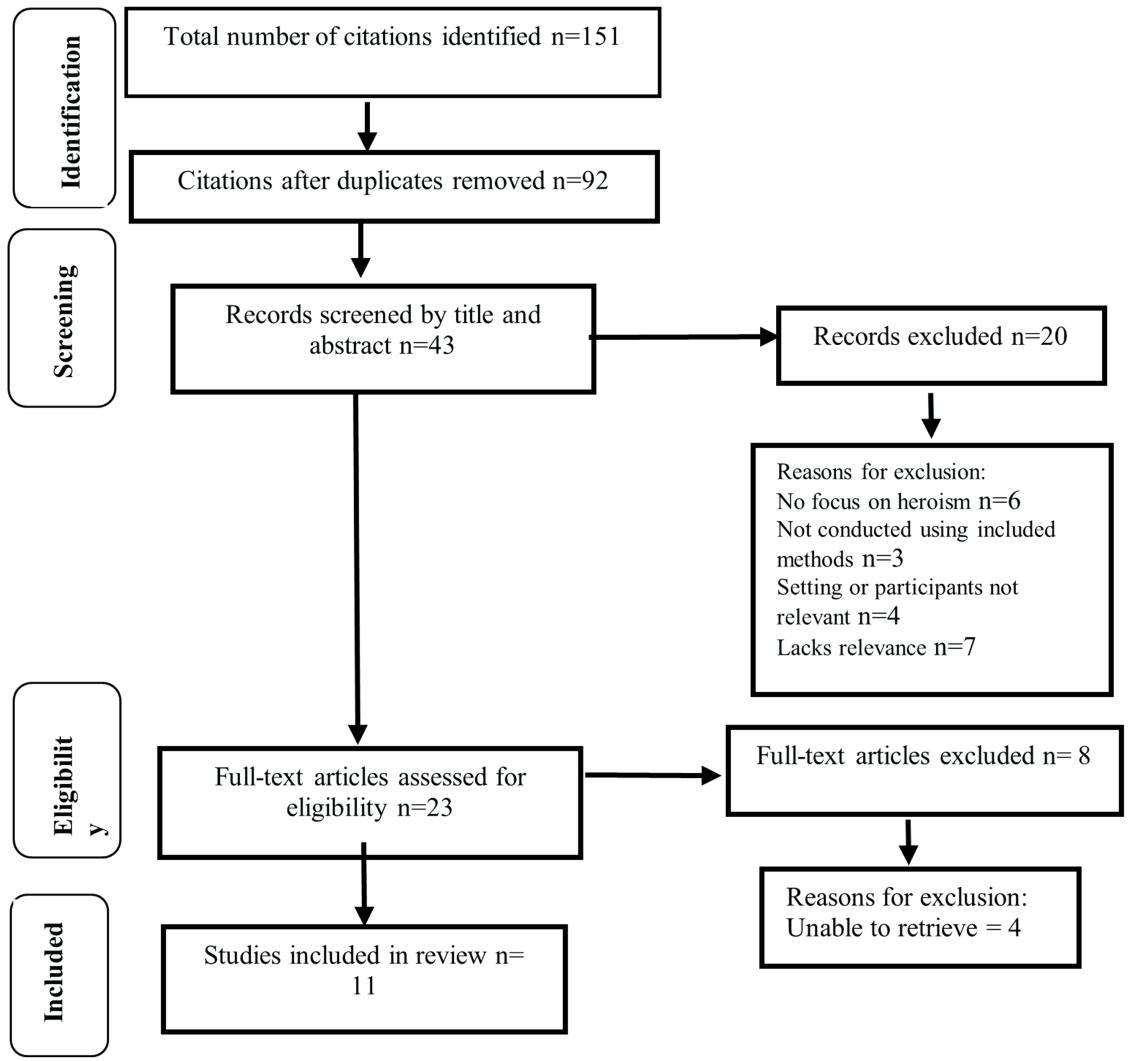
PRISMA-P chart of studies screened and in the final sample.

151 papers were retrieved; eleven were included in the final sample (Figure 1). No studies specifically explored heroism and paramedic practice, five involved heroism within wider paramedic studies and six in relation to non-paramedic health care workers. Studies included in the final sample are presented in Table 1. Quality of studies included two very low, four low and five moderate. Methodologies included post-structural discourse analysis, ethnography, grounded theory, phenomenological analyses, narrative accounts, bricolage methodology using narrative theory, and thematic review. Methods included surveys, in-depth interviews, reviews of literature and media documents, secondary data, and textual analysis, and observational field work. Studies were carried out in United States, France, Canada, Australia, and Sweden.

**Table 1:** Final sample of papers included.

Study	Design	Method of data collection	Method of data analysis	Setting/clinician	Themes	Quality (*Combined CASP/Quality /confidence grade/cerqual)
Hennekam et al. (2020)	Qualitative, inductive techniques	SURVEY AND IN-DEPTH INTERVIEWS	Qualitative inductive coding, NVIVO	Nonphysician health care workers (nurses, hospital attendants, technicians, and other staff who maintain the safety, cleanliness, and administrative functions within hospital) France	COVID-19 enabled visibility of healthcare workers as public heroes. Exploration of 164 non-physician healthcare workers perceive newfound elevated status. Findings that sudden visibility as temporary and treated with scepticism, incredulity, and as devoid of genuinely transformative power. Three themes were identified: The normative experiences of workers’ invisibility before COVID-19 Their interpretation of the sudden valorization of their activities triggered by the crisis, including the perceived temporal nature of the elevated status of their occupation Their reactions to their newfound hero status	Mod
Tangherlini (2000)	Narrative account	Field research conducted over 5 years.	Observational fieldwork	Medics Pre Hospital San Francisco Bay Northern California	Narrative explaining how the stories medics tell play an important part in their tactical engagement with the myriad relationships that structure their daily lives in the organization.	Low
Furness et al., 2021	Archetypal meanings of being a paramedic: A hermeneutic review	Review of literature which followed the recommendations by Smythe and Spence and Reed, Smythe, and Hocking.	A hermeneutic approach informed by Gadamer’s philosophical hermeneutics was used	Paramedics	The following six paramedic archetypes were identified: Paramedic as stretcher bearer Paramedic as hero Paramedic as male Paramedic as stoic Paramedic as clinician Paramedic as storyteller These related broadly to the principles of service, care and stoicism. These archetypes provided glimpses of how the paramedic is theorized both within and external to the profession, as well as gaps related to how the phenomenon of being a paramedic is experienced amid everyday practice situations.	Mod
Nurok and Henckes (2009)	Grounded Theory	Observational fieldwork	Constant comparison	Pre Hospital Paramedics in New York and Paris	Emergency work is shaped by a wide array of values that are not identically prioritized and that compete with each other in a framework that called a “fluctuating economy”. Themes: Social value Technical value: The priority of action Medical and surgical value Intellectual value: the attraction of complexity Heroic value: “the courage to fail” Perfection and competence value.	Mod
Mohammed et al. (2021)	Post-structural discourse analysis	Media Documents reviews	Inductive and Deductive Coding, NVIVO	Nursing in America, Canada & UK	Three main elements of the hero discourse include: 1. Nurses as a “necessary sacrifice” 2. Nurses as “model citizens” 3. Heroism itself as the reward for nurses	Mod
Lazarsfeld-Jensen (2014)	Bricolage methodology using Narrative Theory	Data refresh of two discrete qualitative research projects conducted in 2011 & Additional literature Search	Application of new theoretical frameworks to the analysis of the extant data	Graduate paramedics in Australia	By excluding familiar themes of stigma and bullying relating to either academic or nursing status, we find other cultural symbols. The symbols include rank and longevity, but most importantly, the right to tell a rescue story. Rescue events were highly valued, a rite of passage, and a normative occasion recognized by peers because of the power of stories in heroic myth making. Stories that achieve mythical status in an enclosed working environment, have the ability to silence newcomers who do not possess them, regardless of education or background. The Rescue myth is potent for paramedics as it may be associated with continuing role dissonance.	Low
Smith (2002)	Discussion Article		Reflections and implications for nursing practice	Nursing	Examine the epistemic nature of myth and its relationship to nursing ontology, practice, and knowledge development, and to interpret the myth of the hero journey as it relates to the meaning of health and healing within the unitary-transformative paradigm.	Very Low
Mac Donald et al. (2018)	Thematic Review	Literature Review		Nursing Limited to English papers within the last 10 Years.	The purpose of this article is to provide a thematic review of the literature on heroism in nursing, in order to understand how recent research in heroism science is being, or could be, applied to the nursing discipline. Findings: Gaining a clear understanding of what constitutes a hero and heroism is essential to applying heroism to nursing and to education of students so they are inspired to act courageously. 9 Themes discussed Challenge of defining heroism Types of Heroes Characteristics of Heroes Behaviors distinct from Heroism Making of a Hero Role of Heroes in contemporary Society Everyday acts of Heroism Role of Heroism in Nursing Physical, Social or Everyday Nursing Heroes	Low
Stokes-Parish et al. (2020)	Contemporary discussion article			Nursing	Propose that the hero and angel constructs undermine the professionalism of the nursing workforce, and reinforces the perception that nursing is an innately feminine, nurturing role. We argue that this discourse continues to undermine the continuing endeavors to consolidate nursing’s standing as a profession	Very Low
Brideson et al. (2016)	Institutional ethnography	Literature search of visual and print media images	Textual analysis	*Nursing in Australia*	A textual analysis of the images of flight nursing using the methodology of institutional ethnography reveals a number of themes including the glamorous, the romantic, and the heroic nurse. This study illustrates that the way these nurses are portrayed within popular literature mirrors the Australian cultural ethic of heroic bush pioneer, yet at the same time the work these nurses do is undervalued by various omissions and misrepresentations.	Low
Chandler-Jeanville et al. (2021)	Descriptive phenomenological	Semi-structured interviews.	Abric’s theory of central core	49 front line nurses, including 16 nursing students, and 48 family members	Six paradoxical perceptions were identified concerning the pandemic’s consequences: the Silence Paradox, the Hero Paradox, the Workforce Paradox, the Learning Paradox, the Symbolic Exchange Paradox, and the Uncertainty Paradox.	Mod

The following four interrelated themes were constructed capturing heroism and paramedic practice: (a) *Myth, Folk law, and storytelling in heroism and paramedic practice* (b) *The epic journey of heroism and paramedic practice* (c) *Heroes and zeroes: the fluctuating societal value in heroism and paramedic practice* (d) *Politicisation, and objectification in heroism and paramedic practice.*

### Myth, folk law, and storytelling in heroism and paramedic practice

Myth, folk law, and storytelling have long been the raison d’etre of the paramedic profession (Tangherlini, 2000; Lazarsfeld-Jensen, 2014), many of which promote and sustain the notion of heroism. Campbell and Moyers (2011) argues that such myths can be an epistemic form, way of knowing or understanding, for as meaning seekers and meaning makers, we construct symbolic stories that embody the enduring and universal qualities of our experiences. Lazarsfeld-Jensen (2014) shared the most powerful example of such heroic narrative, during 9/11 where firefighters and paramedics dashed into the Twin Towers and 343 died, resulting in them being the heroes of the early 21st century, and arguably the most tragic and powerful example of heroism in paramedic practice folk law spanning Emergency Medical Systems (EMS) organizations and society.

The media powerfully play out stereotypes through fictional and political accounts of heroic health workers (Tangherlini, 2000; Stokes-Parish et al., 2020). Despite this, they provide little opportunity for emergency personnel such as paramedics to tell their stories in their own words (Tangherlini, 2000). This reflects the reality gap which Brideson argues occurs in heroism between the imagery and fictional writing within the media and invisible health workers, where the public absorbs the created mass media image, rather than their actual experiences (Brideson et al., 2016).

Lazarsfeld-Jensen (2014) tells how storytelling as an element of paramedic collegiality perpetuates rescue stories that are then used to define paramedic work. Furness et al. (2021) also identified the “*Paramedic as hero*” as one of six archetypes in their hermeneutic review exploring meanings associated with being a paramedic. Furness et al. (2021) contended that ambulance work is often depicted in the media as heroic and masculine, focusing on saving lives and rescuing patients from dangerous situations, and argues that such hero paramedic narratives seem to be more prevalent in the context of the combined firefighter/EMS. Dalecke (2020) and Hopkins (2021) concur with such a perspective, acknowledging that aspects of risk, self-sacrifice, and putting oneself in danger for others may often be rare in other health care workers, but key to heroism in roles such as paramedics and firefighters where exposure to risk may be more common.

Lazarsfeld-Jensen (2014) argues that the post 9/11 notion of paramedics as rescuers alongside police and fire fighters has persisted in the literature partly due to Mitchell’s rescue personality theory and critical incident stress debriefing (CISD; Mitchell, 1983; Mitchell, 1988; Mitchell and Bray, 1990; Mitchell and Everly, 1997). Lazarsfeld-Jensen (2014) told how this speculative rescue personality theory has been criticized as it conflates rescue occupations with suggestions of positive personality traits, such as dedication, risk taking, desire to help others, and linked them to the rescuers ability to cope with the appalling events (Wagner, 2005).

Based on fieldwork in EMS in France and the United States, Nurok and Henckes (2009) revealed how the content of heroic storytelling by paramedics often involved cases that provide opportunities to perform technical procedures and prove expertise, such as resuscitation, which they suggest are also those that involve the expression of strong emotions which paramedics most vividly remember and recall when valorizing their skills. Lazarsfeld-Jensen (2014) reflected on such story-telling characteristics of paramedics highlighted by Tangherlini (2000) and the temptation of paramedic lecturers to dramatize practice to inspire the young. Lazarsfeld-Jensen (2014) contends that role dissonance may have emerged through the socialization of paramedics at the point of entry to paramedicine, where they are portrayed as rescuers, and this culture is imported by clinical educators who have not yet developed empirical evidence around the role. Such rescue myths suggest Lazarsfeld-Jensen (2014) can impact on student expectations and later role dissonance due to the importance of these major critical events as rites of passage and are disappointed to find that rescue events are elusive in comparison to caring for the chronically ill and aged (Reynolds, 2009) which may influence high attrition in paramedic practice.

Myth, folk law, and storytelling in heroism have been reported to serve many other purposes for paramedics, including tactical engagement with their employing organization and as a psychological outlet for the emotions engendered by encountering human suffering daily (Tangherlini, 2000). Tangherlini (2000) found that in such stories many paramedics choose to present themselves as anti-heroes, quick for a sardonic quip, and create narratives involving dark humor, casting the horrible into the world of the mundane, which they suggest also helps trivialize, and make less horrific, grisly scenes of suffering, and death. The anti-hero narrative was also found by Furness et al. (2021) and influenced their “*Paramedic as hero*” archetype theme where Hero stories’ were also viewed with derision by paramedics, and rather than self-glorification, they tell heroic anecdotes about their team. Paramedic stories can be deeply moving and personal, reflecting their inability to “do more” that constitutes the major crisis of the event and prompts the narrating, and in this context Tangherlini (2000) suggests that the story allows exploration of the possible range of actions or whether something could have been done differently to reach a positive patient outcome, and thereby eliminating that possibility; they therefore exert a narrative power over an event in which they felt powerless.

### The epic journey of heroism and paramedic practice

The central features of heroism are suggested to include moral integrity, bravery, and self-sacrifice (Kinsella et al., 2015). However, Smith (Smith, 2002) examined the epistemic nature of myth and its relationship to health and healing and drew on Joseph Campbell’s analysis of the myth of the hero journey, which is said to involve the three stages of departure, initiation, and return (Campbell, 1968; Smith, 2002; Campbell and Moyers, 2011). Within this journey the hero starts by separating self from the ordinary rhythms of life, entering new territory, undergoing a series of trials, confronting obstacles to achieve initiation into the unknown, and finally returning to share self or what has been learned on the journey.

The hero journey starts with a call to adventure, beginning with a departure of the hero from their existence in everyday life which can come from within, or be thrust the hero from some external force, compelling them to embark on the adventure. Departure can involve necessary sacrifice, risk, brokenness, and sometimes martyrdom. However, HCW’s such as paramedics and nurses often depart from such a normal existence choosing to provide care in humanitarian and disaster relief situations or overseas in pandemics such as ebola or in war zones. Such individuals have been acknowledged as heroic as they endure personal risks, such as illness or injury, contagion of disease, or the possibility of physical harm through violence, and abuse, yet they continue to care for patients (Schroeter, 2007; Darbyshire, 2011; Mac Donald et al., 2018). These serve as examples of such external forces and transformative processes in which the public association with health work is moved from the mundane and unappreciated to heroic discourse along with exceptional and sudden valorization (Hennekam et al., 2020; Mohammed et al., 2021).

Campbell and Moyers (2011) tell how initiation is defined by ceremony, ritual, or test, where the new knowledge, skills and experiences are acquired through this road of trials, and the hero is on a journey of illumination and coming to know. The heroic stories raised earlier by Lazarsfeld-Jensen (2014) and their role as a rite of passage in paramedicine culture may reflect this context. The hero then surrenders on the road of trials, putting aside “*pride, virtue, beauty and life, submitting to the absolutely intolerable*.” (p. 108; Campbell, 1968). Next comes awakening to the Divine, involving discovering one’s character and destiny, where the hero transcends the rational and goes on to discover love, compassion, and peace. The final part of the journey involves the hero bringing something back from his adventure that will be of benefit to the world. In myths this can be magical substances such as gifts, elixirs, or lessons that will save humankind.

Many of the prototypical action sequences of the hero journey are evident in paramedic narratives, including journeys of brokenness, risk, and acquisition special powers of benefit to the world, supporting health and healing. Within their hermeneutic review exploring meanings associated with being a paramedic, Furness et al. (2021) identified and discussed such mythological attributes of heroes of being capable of great acts of strength and specialist ability within their “*Paramedic as hero*” and “*Paramedic as healer*” archetypes however, these are often grounded in myths which transcends the rational.

### Heroes and zeroes: The fluctuating societal value in heroism and paramedic practice

The role of heroes in contemporary society was revealed as a theme by Mac Donald et al. (2018) which drew on the *Heroes Functions Framework* of Kinsella et al. (2015). This framework recognized that within society heroes serve the three functions of: enhancing or enriching the lives of others; promoting morals and ethics and protecting others from physical or psychological threats. These functions argues Kinsella et al. (2015) correspond to the basic human needs of safety, social belonging, and living a meaningful life in a civil society. Heroes, therefore, offer meaning, hope, wisdom, inspiration, and opportunities for growth to others (Allison and Goethals, 2016). Above all argue Allison and Goethals (2016) hero narratives provide scripts for prosocial action, promoting moral elevation, and inspiring psychological development. Mac Donald et al. (2018) contend that when people engage in hero-worship, they are drawing on the stories of heroes to positively inspire their own lives in cognitive and moral ways and begin to internalize those qualities, building a stronger, and more positive self-identity. Such heroic qualities suggest Mac Donald et al. (2018) can lead to more social heroic actions, providing meaning, inspiration, and guidance in the everyday lives of people.

Popular cultural, societal, and political heroism narratives may reflect a male-dominated bias (Danilova and Kolpinskaya, 2020). Ambulance work and its culture are also often depicted as heroic and masculine (Furness et al., 2021) despite marked workforce demographic changes in recent years. Furness et al. (2021) suggest this is likely due to its military origins and association with other male-dominated emergency services such as Fire Services.

Nurok and Henckes (2009) argue that professionals make decisions based on judgments about a patient’s value, and that emergency work is shaped by a wide array of values that are not identically prioritized, but compete, which they articulated through their “*fluctuating economy*” framework. Nurok and Henckes (2009) reported how performing resuscitation can result in survival or death and the result hoped for was what an American paramedic described as a “*Save*,” which are much publicized and emergency workers vividly remember and recount in detail. Nurok and Henckes (2009) recognized that every resuscitation call represents the opportunity for such “*victory*” and possess “*heroic value*.” Nurok and Henckes (2009) drew on the work of Fox and Swazey (Knox, 1975) who reported how pioneer transplant surgeons similarly thought in terms of “*succeed or fail*” “*victory or defeat*” and suggested that such scenarios have heroic value where emergency actors must have the “*courage to fail*” as articulated by the following quote:

“*I believe that the fact of walking a tightrope attracts us. We need our dose of adrenaline otherwise one is in withdrawal. Working outside in trying conditions, taking care of patients who would certainly otherwise die, performing technical acts, nothing like it to motivate me*” (p. 508; Nurok and Henckes, 2009).

Nurok and Henckes (2009) also found however that some emergency calls possessed no heroic value, and considered to be:

*“the non-valuable dirty “garbage” work that must be waded through in order to perform the desirable valued work of resuscitation”* (p. 508).

Nurok and Henckes (2009) argue that heroic value is linked to social utility, recognition, and self-esteem as it is shaped by the work of resuscitation. Nurok and Henckes (2009) also suggest that heroic value affects how professionals engage in therapeutic decisions, i.e., more aggressively treating young patients, or those in poor health receiving less “*heroic intervention*.” This view of heroic actions and fluctuating economy in paramedic practice may relate to what Sudnow (1967) and Glaser and Strauss (2017) term as social “*worth*” or “*value*” of patients.

The relationship between society and HCW’s such as paramedics was brought into sharp focus during the COVID-19 pandemic, where society, the media and government promoted the NHS Heroes narrative. Hennekam et al. (2020) reported how the media had called attention to such heroism of health care workers and deemed them essential, suddenly making them ‘hypervisible’ as told by one Nurse:

*“The pandemic has made our professions more visible and has highlighted its importance. Health care workers are the ramparts against the virus.”* (Participant 11, Nurse).

Hennekam et al. (2020) highlighted the significance of invisibility of health workers who may feel undervalued, as the feeling that one’s occupation matters has been related to greater self-esteem, lower levels of depression and anxiety, and more positive affect (Rosenberg and McCullough, 1981; Baumeister and Leary, 1995). Hennekam et al. (2020) found that such visibility can change suddenly during crisis periods, and some participants in their study interpreted the public support as recognition of the indispensability of their work for society. Hennekam et al. (2020) subsequently celebrated the heroism narrative calling for change through the social valorization of invisible work of work carried out by all individuals employed in the healthcare sector.

Hopkins (2021) examined aspects of the social role of medicine exposed by the language of heroism and recognized that heroic acts are generally uncommon within a society and are judged on the amount of self-risk that a hero is subjected to (Hopkins, 2021). Hopkins (2021) highlights how paramedics and firefighters are recognized as heroic due to risk inherent in their roles, and the COVID-19 pandemic broadened the heroism narrative to include wider groups, where such risks may usually be relatively rare. Chandler-Jeanville et al. (2021) explored the perceptions and experiences of the COVID-19 pandemic amongst frontline nurses and their relatives and identified six paradoxical perceptions concerning the pandemic’s consequences; one of which was the *Hero Paradox*. Most of the nurses did not consider themselves as heroes, which was consistent with a similar study we conducted with Paramedics (Rees et al., 2021). Nurses in Chandler-Jeanville et al. (2021) considered it as their duty to work during the pandemic and one nurse with a history of cancer said:

*“I thought yes I was more at risk, but that is my job after all.”*

Chandler-Jeanville et al. (2021) found that nurses’ relatives did however consider them to be heroes, which also reflected views of paramedics in Rees et al. (2021) who believed their relatives would consider them as heroes. This view of heroism by nurses relatives in Chandler-Jeanville et al. (2021) and paramedics in Rees et al. (2021) did however reflect the necessary risk discussed earlier as told by one paramedic:

*“I know if I caught it* [COVID-19] *I know my dad would call me a hero”* (Rees et al., 2021).

And a Nurses relative:

*“were proud of them because they put their life at stake in caring for others despite their exhaustion and their difficult working conditions”* (Chandler-Jeanville et al., 2021).

The newfound Hero status of HCW’s was rejected by some participants in Hennekam et al. (2020) due to the perception they were simply doing their work, how they felt objectified, used, and manipulated; and as a defence mechanism out of fear of being hurt, and as they did not believe their elevated status would last, as described by one Nurse:

*“This brutal recognition is appreciated, but it is simultaneously incomprehensible for most of us whose workload and suffering have been ignored by our hierarchy and the authorities for too long. This valorization of our work right now will not last, probably. As soon as society no longer feels in danger, everyone’s interests and the economy will once again become the priority”* (Participant 12, nurse).

### Politicization, and objectification in heroism and paramedic practice

Tangherlini (2000) highlighted that in everyday life for paramedics working in ambulance organizations political pitfalls present, which are often out in the open, due to decentralization of work, and an environment of never knowing where one stands. This they suggest motivates them to frequently consider their position, which they do through their storytelling involving hero narratives. Tangherlini (2000) stated that:

*“Medic storytelling can be seen as a tactical engagement with the organisation-its rules, its constraints, its expectations, its apparatus of surveillance, its rewards and its punishments-as well as with the myriad outside groups that medics encounter on a daily basis, including patients, bystanders, police, firefighters, and hospital personnel”…“Much of this manoeuvring takes place through the storytelling.”*

Brideson et al. (2016) explored images of flight nurses (FN’s) in Australia. Their findings again reflect the politicization and objectification of such Heroism narratives in HCWs. Brideson et al. (2016) reported how the image of romance, glamour, excitement, and adventure of FN’s portrayed within popular fiction and films coincided with the 1940s (WW2), 1950s (Korean War), and 1960s (Vietnam War) wars, and this portrayal of them as heroines of the armed forces was linked to military recruitment drives (Parry, 1997; Vuic, 2010). Brideson et al. (2016) however highlighted how this heroic portrayal was far removed from the reality, as FNs worked hard and long hours, in aircraft not labelled as a medical, carrying dangerous cargos, loading patients into aircraft when under fire from the enemy, and in isolation without medical supervision (FLYING NURSES OF THE R.N, 2022). These issues along with the fatigue and personal issues were however never discussed (Brideson et al., 2016; FLYING NURSES OF THE R.N, 2022).

Politicization and objectification in heroism may also be seen in contemporary narratives. Mohammed et al. (2021) argued that during the COVID 19 pandemic, employing hero worship became a low stakes technique used by politicians and other leaders to convey public messages about idealized citizenship and collective resolve. Mohammed et al. (2021) cites many examples of such objectification of health workers by political groups, including the anti-Trump Lincoln Project (The Lincoln Project, 2020) which produced a YouTube video with images of nurses in protective gear appearing physically exhausted, and anti-lockdown protesters, screaming in front of public buildings. The video included the words:

“*There are two types of Americans that have emerged through this pandemic; Those who sacrifice and those who demand*.”

Mohammed et al. (2021) reflected on corporations, politicians, and the public using such heroization of nurses’ suffering for their own political, economic, and cultural ends, arguing that it amounted to a form of performative allyship, where those with decisional and economic power signal support, but fail to engage in the educational, self-reflexive, policy, and structural changes inherent in more genuine forms of allyship (Erskine and Bilimoria, 2019; Krause and Miller, 2020).

Hall et al. (2003) conducted content analysis of local and national news media documents to describe nursing work life issues as portrayed in the media during the SARS crisis in Toronto. Within this study the theme of ‘*Nursing Virtue: Nurses as Heroes and Professionals*’ was revealed, influenced by the military metaphors of disease prevention and treatment and the battle to be fought highly prevalent in media coverage. Hall et al. (2003) found that nurses and other health professionals were frequently described as Heroes, “Superhumans” and ‘Soldiers fighting the “invisible enemy” where it was reported that: *In a battle, not every hero wears a helmet.* Caption below photo of a nurse wearing a mask, (Hall et al., 2003; Ferguson, 2020). Nurses in Mohammed et al. (2021) however believed differently, and that once the pandemic was over, interest in, and appreciation for their work would quickly fade as told by one Nurse:

*“People have time to applaud us because they are at home. They think of us because they are at home. The government is thinking of us because it has no choice but to throw flowers at us so that we are ready to go to work without flinching. But in a few months, everything will be forgotten. No applause. No salary increase. No revaluation of professions”* (Participant 81, social worker).

Such ambivalence to the heroism narrative has also been reported in paramedics and other HCWs. Tangherlini (2000) reflected ambivalence to such stories of Heroism, telling how:

*“Among the medics in my fieldwork area, I have found a deeply cynical and self-deprecatory storytelling tradition-one that countermands the media representations…. Rather than play into media presentations of them as silent heroes “just doin’ our job,” paramedics tend to present themselves as anti-heroes, always ready with a sardonic quip in even the most horrific situations.”*

And:

“*the occupational storytelling is quite common and is referred to emically as “bullshitting” or “telling lies.”*

Brideson et al. (2016) highlighted the perceived reality gap between imagery and fictional writing verses voices of the nurses, and because of such invisible work, imagery created by mass media is absorbed by the public, rather than communicating the actual experiences of such health workers. Hennekam et al. (2020) suggests an explanation for this, arguing that sudden social valorization of certain occupations does not necessarily trigger changes in self-perception, but rather, they have a choice in how to respond to the change in their social image, and can involve critical reflections of occupational dignity, professional integrity, and societal hypocrisy. Studies appear to reflect such discourse in reconciling Professionalism and Heroism narratives with the media and society, and Nurok and Henckes (2009) highlights the important differences between social value as described by Sudnow (1967) or Glaser and Strauss (2017) and the professional values.

Stokes-Parish et al. (2020) recognized that nurses should possess integrity, compassion, and competence, but questioned the necessity for the Heroic characteristics of bravery and self-sacrifice to be an effective health professional? Stokes-Parish et al. (2020) argue that the Heroism narratives invoke notions of magic, mysticism, the perception of superior courage, or morality which disregard the skill, training, and knowledge underpinning practice and the investment of time, effort, and commitment made. Mohammed et al. (2021) highlighted this discourse, telling how:

*“The hero discourse is not a neutral expression of appreciation and sentimentality, but rather a political, social, and cultural technique employed to accomplish multiple aims such as the normalization of nurses’ exposure to risk, the enforcement of model citizenship, and the preservation of existing power relationships that limit the ability of front line nurses to determine the conditions of their work”* (Mohammed et al., 2021).

## Discussion

Heroism has long been an important part of the paramedic story, despite this, our metasynthesis identified no papers specifically investigating this issue. We did however retrieve studies involving Heroism narratives among paramedics within the wider care contexts and in other HCWs.

Within our metasythesis *Myth, folk law, and storytelling of heroism in paramedic practice* emerged as a theme, and explorations of storytelling amongst such occupations, highlight its important role in the lives of professionals and organizations (McCarl, 1976; McCarl, 1978; McCarl Jr, 1978; Mullen, 1978; McCarl, 1985; Santino, 1990; Tangherlini, 1998; Tangherlini, 2000). Within these stories of heroism the powerful representation of the paramedic rescuer was revealed. Lazarsfeld-Jensen (2014) argued this myth may have emerged through the rescuer personality theory of Mitchell and Bray (1990) and described its use in Critical Incident Stress Debriefing (CISD) employed by organizations to improve mental health and productivity of employees. Mitchell and Bray (1990) suggested rescue personality characteristics of emergency service workers such as paramedics, which included being action oriented and obsessed with high standards of performance and suggested that emergency workers must be aware of “*the unique personalities of emergency personnel and the special jobs they perform*” (p. 43). Rescuer personality theory and its role in CISD (Mitchell, 1983; Mitchell, 1988; Mitchell and Bray, 1990) is controversial and not supported by evidence (Wagner et al., 2009). Conversely, informal de-briefing mechanism and storytelling developed by paramedics may be preferred which serve as a psychological outlet, free from potential management censure implicit in the CISD, which allows for expression of a range of gore or violence beyond what is generally accepted (Tangherlini, 2000).

Paramedic stories reflect societies heroic portrayal of them through demonstration and valorization of special technical powers and abilities, such as their role in in resuscitation. Paramedics also appear however to reject such a portrayal, which presents an important paradox, as heroic stories have been reported to allow exploration of the range of possible actions or if things could have been done differently to reach a positive patient outcome, which Tangherlini (2000) argues allows paramedics to exert narrative power over an event in which they felt powerless. Paramedic heroic stories may therefore reveal tensions and chaos, where, in their attempts to make sense of their experiences, graphic stories, use of dark humor, and presentation of themselves as anti-heroes may reveal efforts to reconcile such Heroic narrative with the realities of their practice and limitations of their role, which contrasts with the myth and folk law that has emerged around them.

Heroism narratives in paramedic practice show echoes of Frank’s (2013) “*wounded storyteller*,” where empathic bonds are created between storyteller and listeners. Frank (2013) defines three different types of narrative which are also reflected in Paramedic Heroic stories and include: the restitution narrative (where the aim of a journey is to get back to where one started from); the chaos narrative (where a journey becomes too painful and confusing to unravel) and the quest narrative (where suffering is met head on and ultimately transforms the person).

Paramedic heroic stories may also reflect moral injury which has been associated with potentially psychologically traumatic events (Nash et al., 2010; Jordan et al., 2017) and can involve cognitive, emotional, spiritual, or existential struggles (Buechner and Jinkerson, 2016; Barnes et al., 2019; Griffin et al., 2019). Paramedics are motivated by a desire to help their patients and community (Lim, 2017) but performing artificial services or interventions to satisfy the expectations of the public has been associated with increased moral distress in paramedics (Bremer and Sandman, 2011). Lentz et al. (2021) identified the impact of performing CPR on a patient who will clearly not benefit as such an unnecessary artificial intervention, which may be related to their strong commitment to help and heal.

Interviews with Swedish ambulance personnel identified that fear of not doing enough for patients evoked feelings of insufficiency and worthlessness (Zhao et al., 1998; Jonsson and Segesten, 2004). Ethical dilemmas can also arise from conflicts between truth telling and protecting the wellbeing of family members, for instance, when Paramedics are faced with deciding to share bad news to family members or lying about the gravity of a patient’s condition in an attempt to protect they from psychological pain or distress (Norberg, 2013; Özcan et al., 2014). Heroism in Paramedic practice may therefore impose unrealistic expectations upon them and influenced by a disconnect between their heroic portrayal in society and mythical powers verses real world tions of their care, which may be creating conditions for moral injury.

Heroic storytelling and myth in paramedic practice may serve as rites of passage, with paramedics, and paramedic educators valorizing skills through such stories to a range of people and groups including developing paramedics (Lazarsfeld-Jensen, 2014). Rescue stories are highly valued, rites of passage, and hold power in Heroic myth making Lazarsfeld-Jensen (2014). However, stories that achieve mythical status can silence newcomers who do not possess them, and may reflect the hero journey, acquisition of special powers, and have been suggested to exist to mark the change of rank from outsider to insider in many different organizations and nations (Østvik and Rudmin, 2001; Groah, 2005; Johnson, 2011).

We reported the hero journey involving the stages of departure, initiation, and return, which may result in necessary sacrifice, risk, brokenness and sometimes martyrdom which is also evident in media depictions and can draw on religious notions of martyrdom to describe HCWs as selflessness in dangerous conditions. We argue that a discursive technique may have emerged in the mass media where Paramedics and other HCWs are positioned as hardworking subjects, which contrasted with “harmful” individuals and groups that denied the severity of the pandemic or resisted public COVID-19 measures. Media outlets also provided a platform HCWs unions who criticized politicians for employing the hero discourse throughout the pandemic, while simultaneously restricting the ability of nurses to bargain for an equitable wage (Ferguson, 2020). This depiction of HCWs as heroes is not new, and in their study of the image of the nurse in mass media, Kalisch and Kalisch (1983) identified that nurses have been portrayed as angels of mercy since the mid-nineteenth century.

Radical alterations in public discourses and attitudes provoked by catastrophic events such as the COVID-19 pandemic can move invisible workers to a position of hypervisibility (Mahalingam et al., 2019). Hennekam et al. (2020) reported the significance in the reality gap and visibility of workers in their study examining sudden Hero status among non-physician health care workers during the COVID-19 Pandemic. They suggest that in healthcare, physicians are highly visible, and hold higher status than nonphysicians, who often regard themselves and their work as invisible (Nembhard and Edmondson, 2006; Allen, 2014). Hennekam et al. (2020) reported how the COVID-19 pandemic radically changed such invisibility by putting all HCWs under the spotlight of public attention and high praise. Whilst this was transient, Hennekam et al. (2020) argued that the outpouring of support for their efforts during the COVID-19 pandemic recast health care staff across all organizational ranks as “essential workers” elevating them all to the position of “heroes” as shared by one study participant:

“*Because of the media and the multiple documentaries on our profession since the outbreak of the COVID-19, people have become more aware of the importance of all the medical and paramedical professions and that, without us, the hospital cannot function* (Participant 68, hospital attendant).

From a societal perspective, Mohammed et al. (2021) however described such a form of Hero worship as being performative, as it is enacted in highly visible ways, appearing legitimate, eliciting self-congratulations, or a “*virtual pat on the back*” (p. 72; Jacobson, 2020).

Our interpretation of the visible and public heroism narrative recognize that it may hold wider societal benefits, reflecting nourishment of the human mind and spirit, offering meaning, hope, wisdom, inspiration and opportunities for growth to others, scripts for prosocial action, promoting moral elevation as described earlier (Allison and Goethals, 2016). Moreover, in such hero admiration or hero-worship, we recognize as with others how they are drawing on the stories of heroes to positively inspire their own lives in cognitive and moral ways and begin to internalize those qualities, building a stronger, and more positive self-identity. Mac Donald et al. (2018) contends that when people engage in such heroic qualities it can lead more social Heroic actions, providing meaning, inspiration, and guidance in the everyday lives of people and promote personal and social development. Provision of pre hospital and paramedic care is however a collaborative exercise involving many groups and individuals. Paramedics therefore have to strike the balance between communicating the contributions of their profession and the impact on healthcare outcomes whilst acknowledging, making visible, and lifting up the role of the public and wider health care team.

Fluctuating social value and politicization in paramedic practice may be reflected in early paramedic programs such as Freedom House which enabled a group of unemployable Black laypeople to establish a model for paramedic training that ultimately set the U.S. standard. At the time, police officers and morticians supplied most prehospital care, which many black Americans relied upon as they could not afford private hospital transport, yet white service operators avoided Black communities who received unfair treatment and inadequate access to medical care (Trotter and Day, 2010; Edwards, 2021). Success of Freedom House resulted in sudden valorization, of their paramedics, some pursued advanced degrees, and leadership roles, but many found themselves unemployed when it was disbanded and a predominantly white “superambulance” service created, jettisoning the social goals of Freedom House by excluding the Black men and women who pioneered EMS standards. Freedom House was dissolved in 1975 (Caroline, 1977).

The COVID-19 pandemic also serves as a contemporary example of the sudden valorization of frontline HCWs being described as heroes (Hall et al., 2003). One of the most recognizable nursing figure of COVID-19 is the Banksy’s acclaimed painting of a “Super-nurse” who is female, masked, wearing a traditional nursing cape and depicted being as specially selected by a small child from a toybox of other superheroes (Einboden, 2020). These Hero depictions give us pause to reflect upon the appropriateness of the moral ideals of health professionals such as paramedics being presented through heroism narratives, which may actually represent an undercurrent that is ever-present in their sense of moral identity (Hall et al., 2003).

The public image of HCWs is, to a large extent, affected by the invisibility of nurses, and the way they present themselves (Hoeve et al., 2014). As the media called attention to the heroism of HCWs who were deemed essential, the pandemic changed the way their work was viewed suddenly made them hypervisible. Paramedics therefore have a difficult balance to strike between communicating the contributions of their profession and the impact on healthcare outcomes whilst acknowledging, making visible, and lifting-up the wider health care team.

Fluctuating social value and ambivalence from paramedics was recently reflected in Rees et al. (2021) during the COVID-19 pandemic where paramedics were portrayed as NHS Heroes in society, while Victory in Europe (VE) day celebrations saw social distancing rules flouted and sharp increases in assaults on emergency staff. Paramedics in Rees et al. (2021) reported that when the pandemic was over, they would be forgotten about by the public and return to long hospital delays, inappropriate use of ambulances and violence and aggression directed towards them.

Heroism is said to include moral courage, which some encourage fostering in health professions as this behavior requires justice values, compassion, the ability to act ethically, effecting change and motivating others by being role models (Lindh et al., 2010; Halmburger et al., 2016). Mac Donald et al. (2018) drew on Zimbardo (2007) when discussing how the positive role of heroism contrasts with *The Lucifer Effect* which is described as the point at which ordinary people first cross the boundary between good and evil to engage in an evil action. According to Zimbardo (2007), good people can be induced into behaving in evil ways. Mac Donald et al. (2018) argues that conversely, it may be possible to understand how to prevent evil actions and encourage people to behave in good ways, kind not cruel, caring rather than indifferent, creative as opposed to destructive and, thus, to be heroes, rather than villains, or bystanders. Franco et al. (2011) also developed Zimbardo’s theory, suggesting the three broad types of hero subtypes of: martial, civil, and social Heroes. Martial Heroism involves physical risk, and is performed particularly by military personnel, Civil Heroism also entails physical risk but is carried out by civilians. Social Heroism is explained as *“heroic action in the service of ideals*,” where social risks, such as loss of social status, career progression, and income, may occur (Franco et al., 2018). Why then would paramedics seek to hold up the physical risks inherent in martial heroism, and civil heroism? or loss of social status, career progression, and income, which may occur in social heroism? We argue that heroism is damaging to the professional identity of paramedics who should work towards mitigating necessary risk inherent in heroism narratives, making them unacceptable whilst holding up Heroic action in the service of ideals in society in the care of others.

Low acuity clinical presentations dominate paramedic daily workloads (Rosser, 2020), where older people who have fallen account for 6–8% (Simpson et al., 2017; McManamny et al., 2022), and people with mental health problems can account for up to 10% (Shaban, 2005). In contrast, studies have observed that paramedics attend an average of less than two cases of OHCA per year in some areas (Dyson et al., 2015), and whilst studies have struggled to demonstrate association between paramedic experience and survival in treatment of cardiac arrests (Gold and Eisenberg, 2009), survival rates are highest amongst those who received bystander CPR and public access defibrillation (Hawkes et al., 2017). On a population level it may be argued that the paramedic provides minimal impact on survival from OHCA in comparison to early recognition, CPR and defibrillation. Indeed the highest bystander CPR rates are in Scandinavia, where CPR training in schools has been mandatory for decades (Wissenberg et al., 2013) and the World Health Organization (WHO) “*Kids Save Lives*” Statement (Böttiger and Van Aken, 2015) recommends 2 h of CPR training annually in schools worldwide. Harnessing the hero narratives in this context and providing scripts for prosocial action and promoting moral elevation, presents rational and conceptual opportunities for saving more lives from OHCA. In this sense, heroism offers meaning, hope, inspiration, wisdom, and opportunities for growth to others (Allison and Goethals, 2016).

Studies have problematized the hero narratives for health professionals by examining the effects on professional, social, and political identities (Einboden, 2020; Morin and Baptiste, 2020). Stokes-Parish et al. (2020) acknowledged that nurses should possess integrity, compassion, and competence, but questioned whether the characteristics bravery and self-sacrifice are indeed necessary to be an effective health professional? Stokes-Parish et al. (2020) also contend how words that invoke notions of magic or mysticism or the perception of superior courage or morality used in heroism disregard the skill, training, and knowledge underpinning practice. Stokes-Parish et al. (2020) therefore proposes how such harmful commentary of Heroism creates the unintended consequence of undermining professionalism and serve to disempower and silence (Stokes-Parish et al., 2020). The failure of the hero discourse to acknowledge the emotional, psychological, moral, and physical stressors of pandemic work has implications for the future expectations placed on nurses and other HCWs as the crisis continues.

Heroism narratives therefore serve a range of purposes and varying interests for groups and individuals. Hopkins (2021) examined aspects of the social role of medicine exposed by the language of Heroism and reflects how the moral and social identifiers of heroism are constructed, where truly heroic acts he tells are relatively uncommon within a society and judged on the amount of self-risk that a Hero is subjected to (Hopkins, 2021). A paramedic in Rees et al. (2021) told how:

*I do not think it is right that we are posing in masks, and I mean the medical professional as a whole, dancing up and down the corridors meters away, potentially from ITU where people are fighting for their lives… you would not get a crematorium bloke doing a song and dance in a crematorium would you* (P10).

And

*“once it’s over they will forget about us again and it will be back to abusing people again and sitting around in hospitals and where has this been until now when you needed us”* (P14).

Few would argue that “*crematorium blokes*,” or indeed mortuary attendants or cleaners are exposed to less risk than paramedics, nor how vital their roles are to society. Such circumstantial ascribing or heroism status reflects Foucault (1980) reminder to us that each discourse has its own politics of truth and untruth, which, through practices of exclusion, distinguish what some consider to be true and false knowledge. Foucault (1980) argues that certain discourses are dominant (for example, medicine, experimental science, capitalism, etc.) because they are largely accepted and employed to “shut down” the possibilities of writing, speaking, and thinking in ways that challenge this authority (Hook, 2001). Through discourse, social actors can both constitute, and ensure the reproduction of, existing and dominant social systems, using different forms of knowledge selection, exclusion, and domination (Hook, 2001). In addition, discourses exist as various practices, also known as discursive practices, which Foucault (1980) claims “*systematically form the object of which they speak*” (p. 49) and shape how people and groups act, think, behave, and act.

Prehospital care is quite different from the other occupations in healthcare system and heroism discourses are played out within the context of many ethical, psychological, welfare, educational, financial, and administrative factors which have been shown to affect occupational motivation in staff (Sheikhbardsiri et al., 2018; Mohammadi et al., 2021). Pre-hospital professionals such as paramedics are also exposed to numerous emotional and care stressors, when working in dangerous conditions, and such high workloads (Sheikhbardsiri et al., 2020; Sahebi et al., 2021; Sheikhbardsiri et al., 2022). Our study is one of many possible constructions of heroism in paramedic practice and the method used may be limited by its lack of objectivity. We however propose that this is its strength, as rich insights have been provided which need further investigating.

## Limitations

We used systematic methods to identify and appraise published research, finding limited literature related to heroism and paramedic practice. Our metasynthesis, therefore, relied on findings across practice and professional contexts. It is also possible that key works may have been missed, such as dissertations and papers which may have involved heroism interchangeably with other terms and contexts. The metasyntheses methodology used also risks synthesizing work from varying philosophical or theoretical perspectives, which may be deemed incompatible, and we therefore recognize that our interpretations should be read with caution. We also recognize that interpretations were made through a paramedic lens as the main researcher was a paramedic, and this may influence the findings. We do however contend that the insiderness of the paramedic is a major strength and compatible with the constructivist approach employed, where along with the transparency of our methods and analysis, trustworthiness can be assured for readers.

## Conclusion

We found no studies explicitly exploring heroism in paramedic practice, we did however include studies involving heroism within wider contexts. Our metasynthesis is therefore based on a very small body of knowledge and is an incomplete representation of heroism in paramedic practice. We have however constructed a metasynthesis which reflects how heroism is ontologically inseparable from the paramedic story and has subsequently entered into their folk law and myths, propagated and adopted by paramedics, organizations, the public, politicians, and wider society. Exploring the story of heroism in paramedic practice provides an epistemic opportunity for knowing, understanding, and acquiring knowledge that is enduring, giving a sense of continuity with the past, present, and future paramedic journey across time, cultures, and its progeny. Exploring the story of heroism in paramedic practice provides reflexive opportunities to consider the position of paramedics in healthcare and society and pedagogically construct professional knowledge.

Much can be learned by reflecting on the prototypical action sequence of the hero journey, of separation from the ordinary rhythms of life, undergoing trials, initiation into the unknown, and returning to share self or what has been learned on the journey. The notion of Heroism in this context raises many questions for the paramedic profession, such as does it place unrealistic demands on all paramedics to be heroic and create an acceptance of risk within their profession? We presented the example of the heroic technical skills in resuscitation and opportunities for ‘saves’ in OHCA, and how these were often grounded in myths which transcends the rational. What then of the overwhelming majority of the paramedic case-mix which lacks heroic value, such as those with mental health issues, or older people who fall for example? What does it tell of the possession of ‘special powers’ as opposed to those acquired though education, learning, experiential, and professional development?

We argue that the notion of heroism in paramedic practice where certain patient groups and skills sets possess heroic value or are rites of passage at the expense of more routine work is problematic and may undermine paramedic professional values. Our metasynthesis highlights that fluctuating social value across their case-mix, politicization, objectification and subsequent ambivalence in heroism and paramedic practice, is as old as the story of paramedicine itself, reflected in early paramedic programs of the 1970’s, and sustained over half a century later in the context of the COVID-19 pandemic. Heroism in paramedic practice vias into the truth-fantasy where, as with other myths, they may not reflect everyday experiences in the world but rather, reveal central truths related to human experience that are beyond this discursive language.

The risks inherent in the paramedic role and that of other emergency rescuers appears to be central to their characterization as Heroes. The COVID-19 pandemic however broadened the heroism narrative to include wider groups who may have otherwise been invisible and where such risks are relatively rare. Heroic social valorization of such invisible work appears to have broken down much of the established order of occupational worth by stressing the indispensability and significance of work carried out by all individuals employed in the healthcare sector during the COVID-19 pandemic. However, this may be premised on the necessary risk, self-sacrifice, and desire to help others in heroism which has been deemed essential in paramedic practice to enable rescuers to cope with the appalling events witnessed in speculative rescuer personality theories. We argue that such heroism narratives in paramedic practice involving normalization of exposure to risk and upholding them as outstanding moral subjects who possess mystical heroic powers may stifle important professional discussions around safety, education, and limit their ability to determine the conditions of their work and reinforce myths around the effectiveness of their paramedic practice at the expense of the broader case-mix.

Our metasythesis did however recognize how such Heroism narratives provide scripts for prosocial action, promoting moral elevation, and inspiring others. We therefore forward the notion that employing such heroism narratives in encouraging people to learn CPR, especially kids could result in saving more lives from OHCA. We also recognize that contemporary pre hospital and paramedic care is a collaborative exercise, involving many groups and individuals, and therefore, harnessing the prosocial aspects of heroism narratives across the wider NHS and society in general may result in improved care.

The stories and mass media image of heroism in paramedicine are absorbed and promoted in society and by organizations, politicians, and the media which may not reflect actual experiences. Despite the significance of heroism in paramedic practice, we suggest that it represents a notable gap in the literature and paramedic professional knowledge which requires further research. Finally, we argue that the heroism narratives in paramedic practice places unrealistic demands on them, and the stories they tell may reflect discourse of wounded, rather than heroic storytellers. Future studies involving qualitative methodologies should focus on this discourse in paramedic practice.

## Author contributions

NR, TA, CH, LS, and JW designed the study, advised on materials and methods, directed its curation, and drafted the final manuscript. NR and TA collected and analyzed data. All authors contributed to the article and approved the submitted version.

## Funding

We have not received a grant or other funding for this research from any agency in the public, commercial or not-for-profit sectors.

## Conflict of interest

The authors declare that the research was conducted in the absence of any commercial or financial relationships that could be construed as a potential conflict of interest.

## Publisher’s note

All claims expressed in this article are solely those of the authors and do not necessarily represent those of their affiliated organizations, or those of the publisher, the editors and the reviewers. Any product that may be evaluated in this article, or claim that may be made by its manufacturer, is not guaranteed or endorsed by the publisher.
